# A new species of *Acartia* (Copepoda, Calanoida) from the Philippines, based on morphological and molecular analyses

**DOI:** 10.3897/zookeys.814.24601

**Published:** 2019-01-08

**Authors:** Khwanruan Srinui, Susumu Ohtsuka, Ephrime B. Metillo, Masahide Nishibori

**Affiliations:** 1 Institute of Marine Science, Burapha University, Muang, Chon Buri 20131, Thailand Burapha University Chon Buri Thailand; 2 Graduate School of Biosphere Science, Hiroshima University, 1-4-4 Kagamiyama, Higashi-Hiroshima 739-8528, Japan Hiroshima University Higashi-Hiroshima Japan; 3 Department of Biological Sciences, Mindanao State University – Iligan Institute of Technology, Iligan City 9200, Philippines Mindanao State University Iligan City Philippines

**Keywords:** *
Acartia
*, Calanoida, mitochondrial genes, Philippines, phylogeny

## Abstract

A new species of Acartia (Odontacartia), A. (O.) edentata**sp. n.**, was collected from Leyte Island in the Philippines. Morphologically, the new species resembles A. (O.) pacifica Steuer, 1915. The female of the new species differs from other species of the A. (O.) erythraea Giesbrecht, 1889 species group in the absence of a pair of sharp spines on the posterior border of the genital double-somite and absence of setules on the lateral margins of urosomites 1–3. Unlike other congeners of the species group, males of the new species lack fine setules along the posterior margin of the prosome. Comparison of the new species with A. (O.) pacifica by pairwise distance data for the 16S (282 bp) gene indicates that these species differ by 20–21%, while the COI gene (636 bp) indicates a difference of 16–17%. The new species seems to be a coastal, occurring in warm waters having a salinity of 33.5.

## Introduction

The planktonic calanoid copepod genus *Acartia* Dana, 1846 so far comprises 64 species worldwide (Razouls et al. 2018). The genus consists of seven subgenera: A. (Acanthacartia) Steuer, 1915, A. (Acartia) Dana, 1846, A. (Acartiura) Steuer, 1915, A. (Euacartia) Steuer, 1915, A. (Hypoacartia) Steuer, 1915, A. (Odontacartia) Steuer, 1915, and A. (Planktacartia) Steuer, 1915 (Boxshall and Halsey 2004; Razouls et al. 2018). The subgenus Odontacartia is widely distributed in brackish to oceanic waters of the Indo-West Pacific and currently accommodates 12 species (Ueda and Bucklin 2006; Razouls et al. 2018). Generally, these can be distinguished by sexual dimorphic features of the posterior prosome, urosomites, antennules, and fifth legs.

The *centura* and *erythraea* species groups have so far accommodated 7 and 5 species, respectively, with the unassigned species A. (O.) lilljeborgi Giesbrecht, 1889. The *centrura* species group now accommodates the following seven species (Steuer 1923; Ueda 1986): A. (O.) bowmani Abraham, 1976; A. (O.) centrura Giesbrecht, 1889; A. (O.) edentata sp. n.; A. (O.) mertoni Steuer, 1917; A. (O.) ohtsukai Ueda & Bucklin, 2006; A. (O.) pacifica; and A. (O.) spinicauda Giesbrecht, 1889.

The common A. (O.) pacifica has a wide distribution on the coasts of the Indo-West Pacific and in East Asian continental waters. The population in the brackish Ariake Sea, western Japan, was identified as a different species, and, after detailed morphological and molecular analyses, was described as A. (O.) ohtsukai Ueda & Bucklin, 2006 and considered to be a continental relict (Ueda and Bucklin 2006).

During our copepod surveys in southeastern Asia, we found an undescribed species of A. (Odontacartia) from Leyte Island, the Philippines. It is closely related to A. (O.) pacifica, but unique in lacking paired posterodorsal pointed processes on the female genital double-somite. Following Ueda and Bucklin’s (2006) methodologies, we are able to define the new species described herein. A key to species of the subgenus Acartia (Odontacartia) is also provided.

## Material and methods

### Morphology and sampling

**Figure 1.**
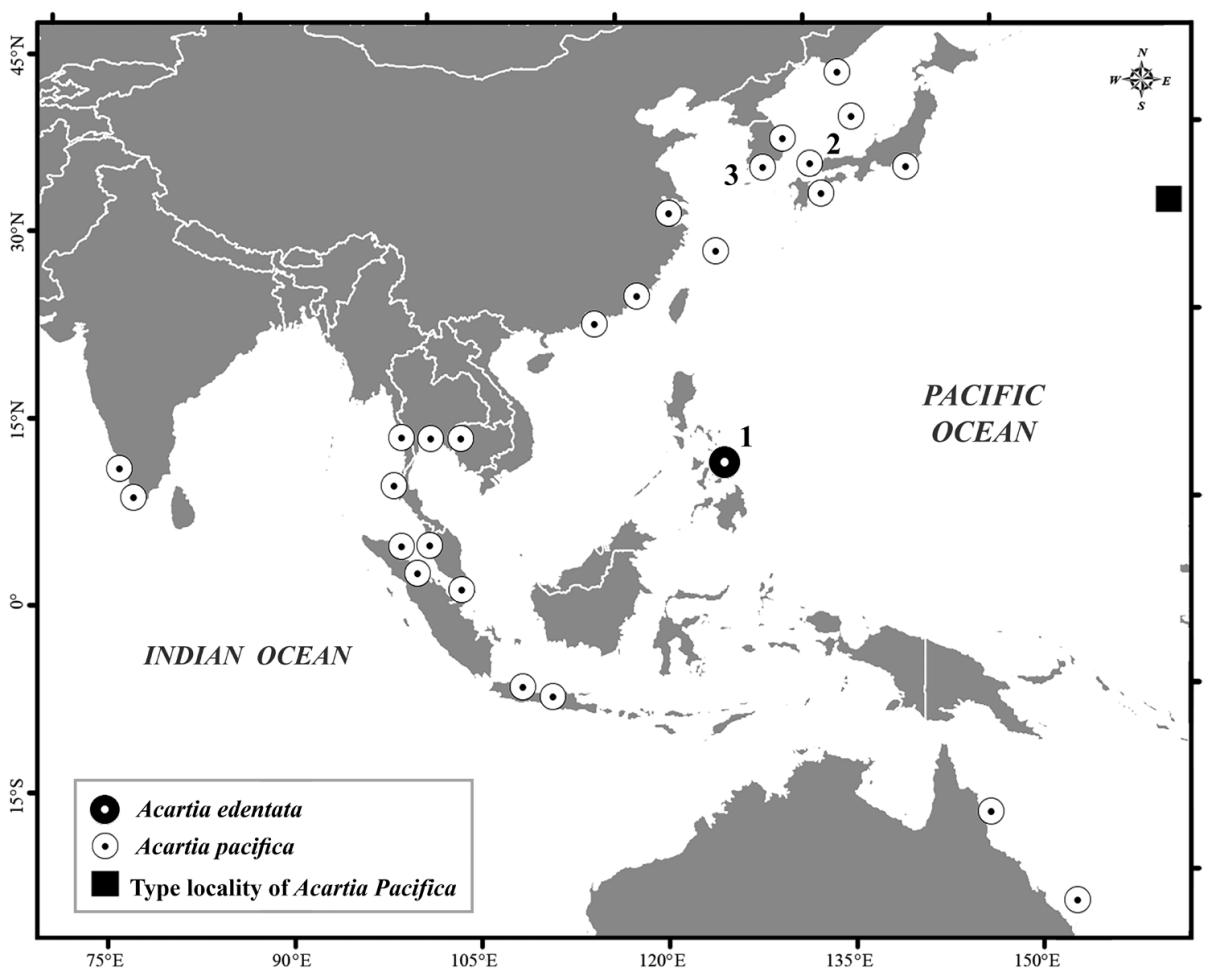
Distribution of Acartia (Odontacartia) pacifica and its sibling species based on data from this and previously published reports. In the present study, samples were obtained from three sites: Station 1, Acartia (Odontacartia) edentata sp. n., Leyte Island, the Philippines (black donut); Station 2, A. (O.) pacifica, Ariake Bay in the Seto Inland Sea, Japan (white donut); and Station 3, A. (O.) pacifica, South Korea Sea, Korea. Data from earlier studies indicate the distribution of A. (O.) pacifica in the Pacific Ocean (Steuer 1915; Tanaka 1965; Brodsky 1967; Ueda and Bucklin 2006); Korean waters (Kang 2011); the Yellow Sea, Chiekong River, Juilong Estuary, Changjiang Estuary, China (Shen and Lee 1963; Chen and Zhang 1965; Shang et al. 2007; Gao et al. 2008); eastern Indonesian waters of Java Sea, Bintulu coast, Indonesia (Früchtl 1923; Mulyadi 2004; Johan et al. 2013); the Gulf of Thailand (Pinkaew 2003); the Indian Ocean (Sewell 1933; Wellershaus 1969; Pillai 1971; Resai et al. 2004; Phukham 2008; Treeramaethee et al. 2013); water of the Great Barrier Reef, Moreton Bay, Australia (Farran 1936; Greenwood 1978); and type locality of A. (O.) pacifica (black square).

The material examined was collected from three sites: Carigara Bay, Leyte Island, the Philippines (11°30'70"N; 124°69'01"E, depth 15 m) during the daytime on August 23, 2013 (local time 15:55); Ariake Bay, Seto Inland Sea, Pacific Ocean (34°18'40"N; 132°56'40"E, depth 12 m) on August 11, 2011 (local time 13:30); and South Korea (34°40'20"N; 127°48'24"E, depth 24 m) on August 30, 2011 (local time 15:00) (Stations 1–3, respectively, in Fig. 1). Water temperature and salinity were measured using a water quality meter, YSI model Pro2030. All specimens were obtained using a series of vertical tows from the bottom to the surface of the water with a conical plankton net (diameter: 30 cm, mesh size: 0.33 mm). Specimens for morphological examination were preserved with a 4% neutral buffered formalin/seawater solution, while those for DNA analysis were fixed in 99.5% ethyl alcohol. Adult acartiids were sorted from the original samples under a stereomicroscope (Olympus SZX16, Olympus, Tokyo, Japan). Specimens were dissected using a stainless steel pin (no. 00), and transferred to a polyvinyl lactophenol solution. Morphological features were measured directly with an ocular micrometer, and were drawn using a camera lucida attached to a compound microscope (Olympus BX53, Olympus, Tokyo, Japan). Male and female urosomites of the new species were examined with a scanning electron microscope (JSM-6510LV, Jeol Ltd, Tokyo, Japan). Terminology follows Huys and Boxshall (1991). Specimens of the species of *Acartia* and A. (O.) pacifica examined in the present study are deposited in the Institute of Marine Science, Burapha University (BIMS–Zoo–0266).

The structure of female and male antennules follows a pattern of the basically uniramous 28 segments. The antennules of both sexes are similar except for the geniculate right antennule in calanoid copepods as in the copepodid I (CI) setation pattern (Boxshall and Huys 1998). We followed Ueda and Bucklin (2006) because the new species more closely resembles A. (O.) pacifica in having equal antennule segment numbers, a similar setation pattern in right antennules of females and males, except segment 5 (XIII), and three rows of spinules ventrolaterally on the second somite of males.

### Molecular analysis

In this study, we used adults of the undescribed species Acartia (O.) from the Philippines and A. (O.) pacifica individuals for genetic analysis of the mitochondrial cytochrome oxidase I (COI) and 16S rRNA (16S) genes. DNA for PCR amplification was prepared from individual males or females placed in microcentrifuge tubes with 50 µl chelex 5%, 1 µl Proteinase K (20 mg/ml). Tubes were heated to 65 °C for 1 hour, boiled at 100 °C for 8 min, and centrifuged at 10,000 *x g* for 8 min. PCR reagents included 5 µl of 10× buffer, 4 µl of 50 mM MgCl_2_, 5 µl of 2 mM dNTPs, 0.25 µl of 10 µM primer solutions, 0.25 µl Taq DNA polymerase (Product no. PL1202, Vivantis, Malaysia) and 30.25 µl distilled water, following (Ueda and Bucklin 2006). We used the universal COI primers COI–LCO1490F: 5’– GGTCAACAAATCATAAAGATATTGG–3’ and COI-HCO 2198R: 5’– TAAACTTCAGGGTGACCAAAAAATCA–3’ according to (Folmer et al. 1994). Primers used for 16S amplification were 16S–167F: 5’– GACGAGAAGACCCTATGA/AG–3’ and 16S–BR–HR: 5’–CCGGTTTGAACTCAGATCATGT–3’ (Palumbi 1996; Bucklin et al. 1998). The PCR amplification protocol consisted of 40 cycles of denaturation at 94 °C for 1 min, annealing at 45 °C for 2 min, and extension at 72 °C for 3 min. PCR products were electrophoresed on a 1% agarose gel to confirm their size and quality, and then purified using the Hiyield^TM^ Gel/PCR Fragments Extraction Kit (PG-913-12041, RBCBioscience, Taiwan). The purified PCR products were sequenced by Macrogen Inc. (Seoul, Korea) with an Automated Sequencer (model ABI 3730 XL, Applied Biosystems, USA).

DNA sequences were manually edited using Sequence Scanner version 1.0 (Applied Biosystems) and compared with the GenBank: A. (O.) pacifica (accession number DQ071177 for COI and DQ071175 for 16S); A. (O.) ohtsukai (accession no. DQ071177 for COI and DQ071176 for 16S); Acartia (Acanthacartia) tsuensis Itô, 1956 (accession no. KC287427 for COI); and A. (O.) erythraea (accession no. DQ320504 for 16S). Sequences and multiple alignments were constructed with BioEdit version 7.1 (Hall 1999). Pairwise distances were determined with MEGA 6 (Tamura et al. 2013) using the maximum likelihood (ML) and bootstrapping 1,000 times (Saitou and Nei 1987).

## Systematics

### Order Calanoida Sars, 1903

#### Family Acartiidae Sars, 1903

##### Genus *Acartia* Dana, 1846

###### Subgenus Acartia (Odontacartia) Steuer, 1915

####### Acartia (Odontacartia) edentata

Taxon classificationAnimaliaCalanoidaAcartiidae

Srinui, Ohtsuka & Metillo sp. n.

http://zoobank.org/2EC6BED6-0611-465D-A0A3-643511DF8542

**Figure 2.**
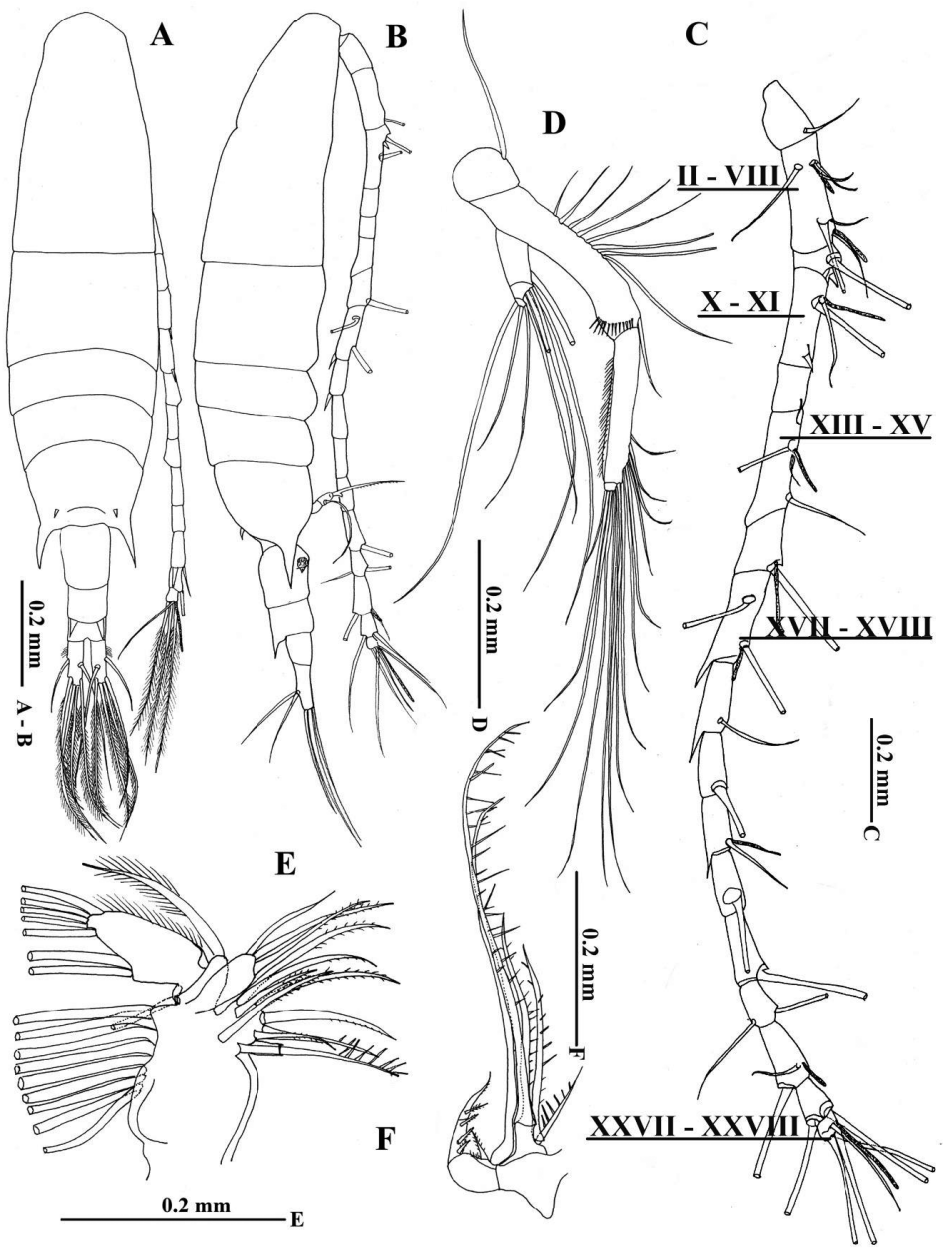
Acartia (Odontacartia) edentata sp. n. female (holotype) **A** Habitus, dorsal view **B** Habitus, lateral view **C** Antennule, Roman numerals denote segment numbers **D** Antenna **E** Maxilla **F** Maxilliped.

**Figure 3.**
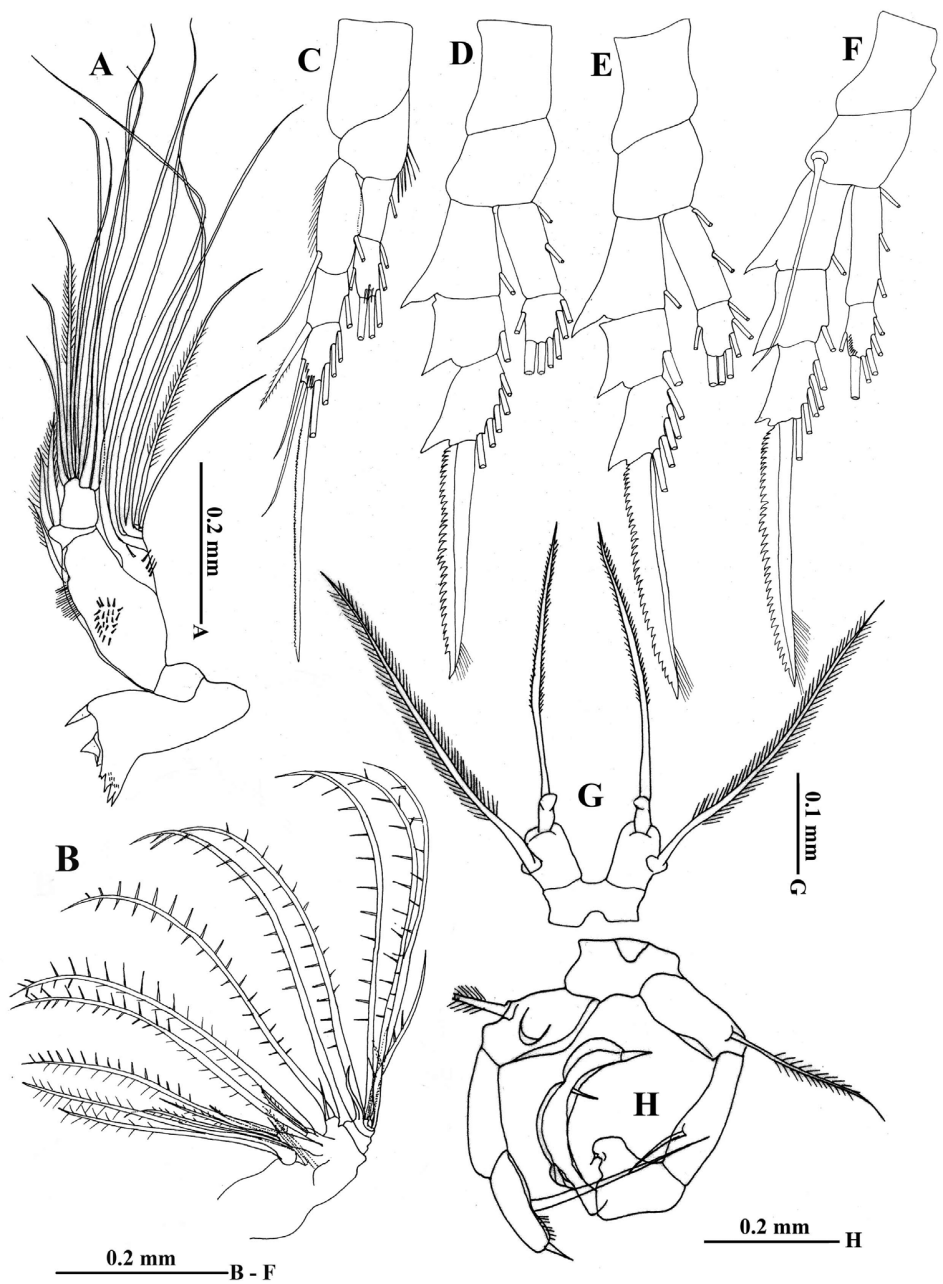
Acartia (Odontacartia) edentata sp. n. female (**A–G**) and male (**H**) (holotype) **A** Mandible **B** Maxilla **C** Leg 1 **D** Leg 2 **E** Leg 3 **F** Leg 4 **G** Legs 5 **H** Legs 5.

**Figure 4.**
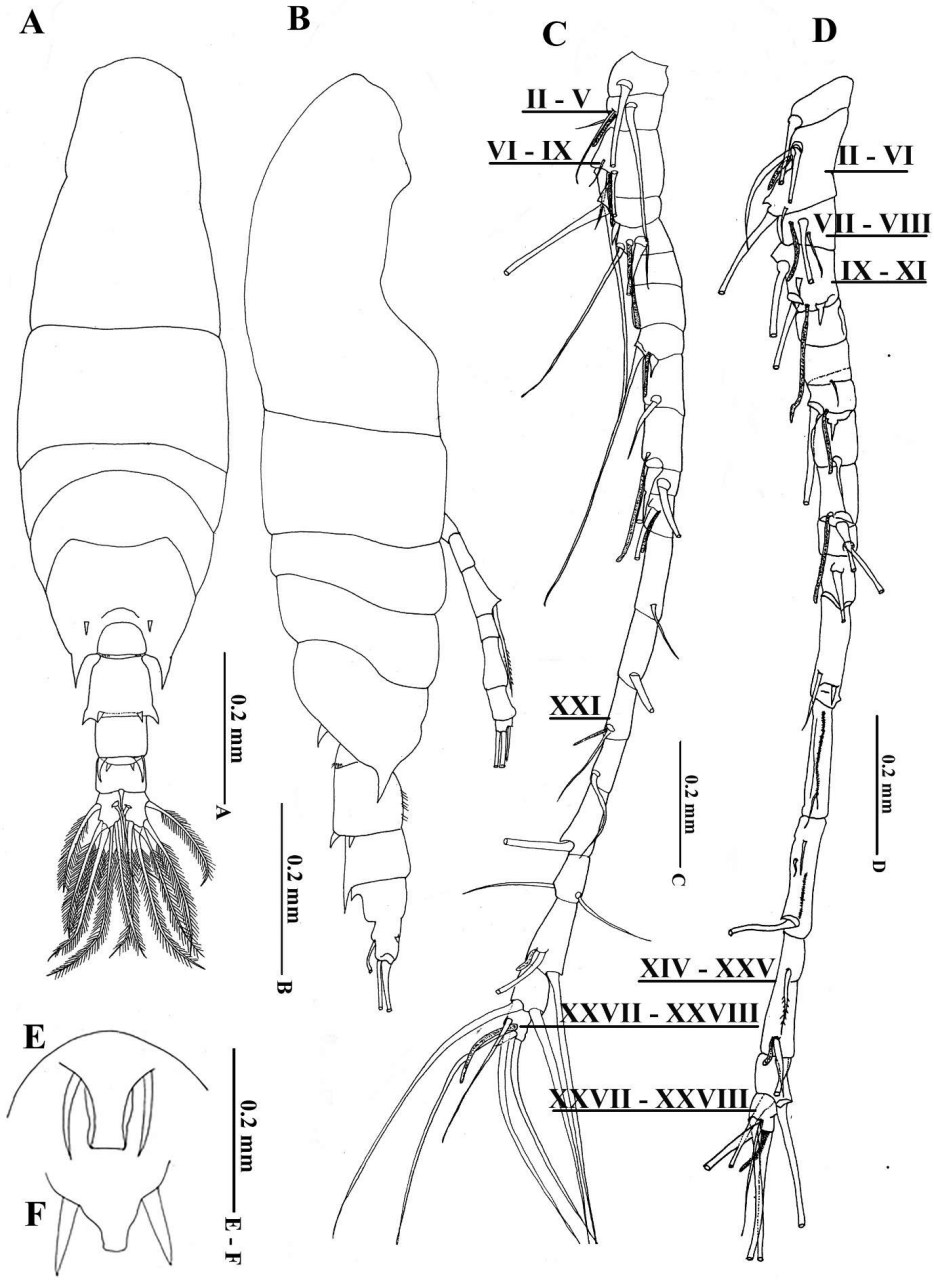
Acartia (Odontacartia) edentata sp. n., female (**F**) and male (**A–E**) (allotype) **A** Habitus, dorsal view **B** Habitus, lateral view **C** Antennule, Roman numerals denote segment numbers **D** Antennule, Roman numerals denote segment numbers **E** Rostrum of male **F** Rostrum of female.

**Figure 5.**
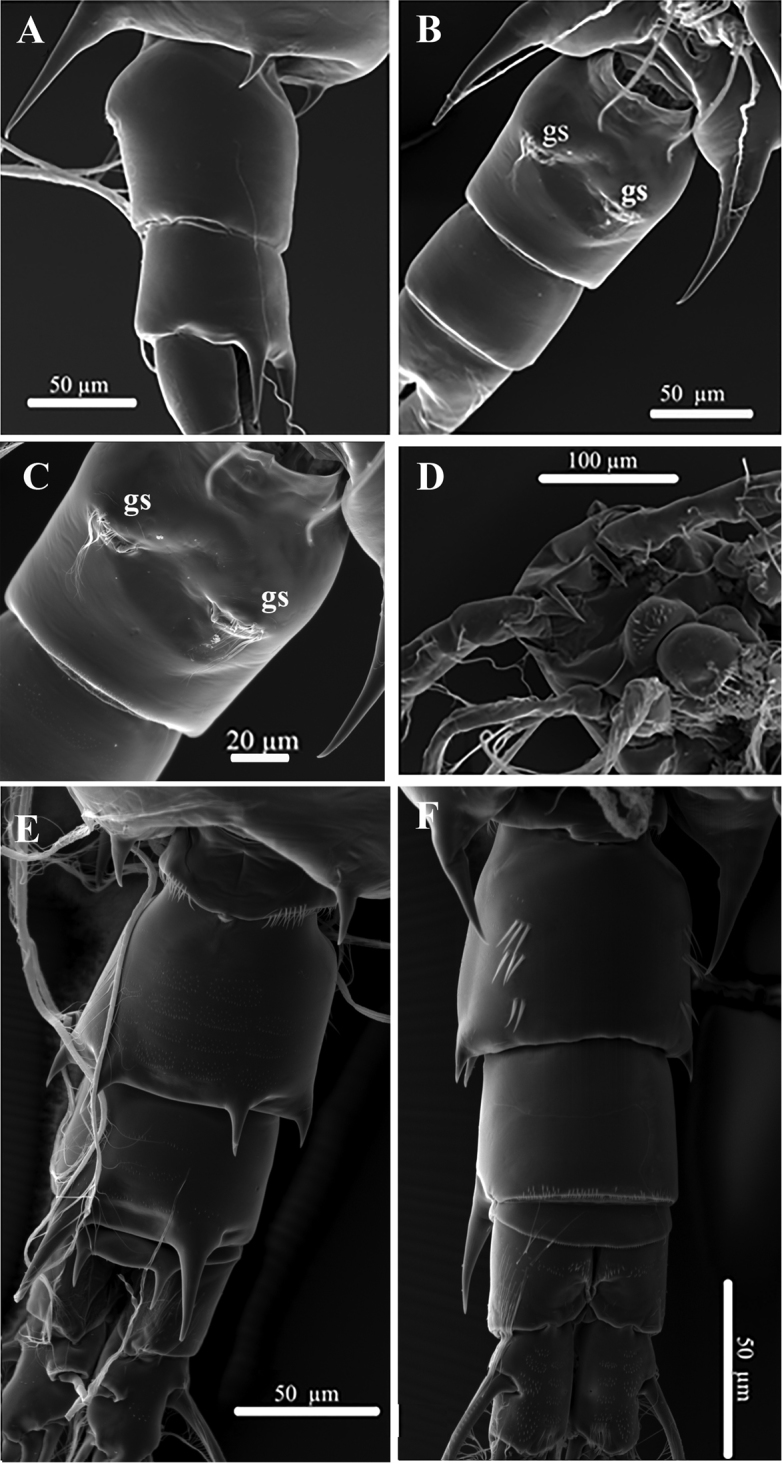
SEM micrographs of Acartia (Odontacartia) edentata, sp. n., female (**A–D**) and male (**E, F**) **A** The genital double-somite lateral view **B** Urosomite, ventral view **C** Genital double-somite, ventral view **D** Rostrum, ventral view **E** Urosomite, dorsal view **F** Urosomite, ventral view.

Figures 2 , 3 , 4 , 5

######## Material.

Type locality: Carigara Bay, Off Leyte Island, the Philippines (11°30'70"N; 124°69'01"E) (Fig. 1), August 23, 2013 (10 ♀, 10 ♂).

######## Types.

Holotype: ♀, dissected and mounted on 2 glass slides (BIMS–Zoo–0267); paratype (allotype): 1 ♂, dissected and mounted on 2 glass slides (BIMS–Zoo–0268); additional paratypes: 4 ♀, 3 ♂ partially dissected and mounted on 3 glass slides (BIMS–Zoo–0269).

######## Measurements.

Female. Total length, 1.19–1.23 mm (mean ± SD = 1.21 ± 0.01 mm, *N* = 10; holotype, 1.19 mm); prosome length, 0.42–0.46 mm (0.44 ± 0.01 mm; holotype, 0.44 mm); prosome width, 0.24–0.29 mm (0.26 ± 0.01 mm; holotype, 0.25 mm). Male. Total length 1.08–1.15 mm (mean ± SD = 1.10 ± 0.02 mm, *N* = 10; allotype, 1.10 mm); prosome length, 0.39–0.41 mm (0.40 ± 0.00 mm; allotype, 0.40 mm); prosome width, 0.23–0.26 mm (0.24 ± 0.01 mm; allotype, 0.25 mm).

######## Descriptions.

Female. Body (Fig. 2A, B) elongate; cephalosome completely separate from first pedigerous somite; anterior margin of cephalosome triangular in dorsal view; rostrum with pair of thick, strong and sharp filaments (Figs 4F, 5F); fourth and fifth pedigerous somites fused. Posterior prosome symmetrical with pair of acute processes on each side: large ventrolateral, pointed processes with pair of small prominences between and pair of smaller, pointed processes dorsally (Fig. 2A). Urosome composed of three free somites; genital double-somite symmetrical with ratio of width–length ratio approximately 1:1, lacking posterodorsal pointed processes (Figs 2A, 5A); second urosomite with pair of strong, posterodorsal, pointed processes; anal somite as wide as long, without lateral rows of fine setules. Proportional lengths of urosomites and caudal ramus 41:22:15:22 (= 100). Caudal rami with setules along lateral margin, and symmetrical with 6 plumose setae (II–VII). I absent, V longest, and VII inserted anterodorsally.

Antennule (Fig. 2C) reaching beyond posterior end of second urosomite, symmetrical, 17-segmented; segments II–VIII completely fused; segments II, IV, VII, VIII, XI, XV, and XVII with aesthetasc (ae). Fusion pattern and setal elements as follows (Roman numerals represent ancestral segments): I = 1, II–VIII= 7 + 2ae, IX = 1+ (1 spiniform), X–XI = 2 + (1 spiniform) + ae, XII = 1, XIII–XV = 3 + ae, XVI= 1 + ae, XVII–XVIII = 2 + (1 process) + ae, XIX = 1 + (1 process), XX = 1, XXI= 1 + (1 process) + ae, XXII = 1, XXIII = 1, XXIV = 2, XXV = 2 + ae, XXVI = 2, XXVII–XXVIII = 4 + ae.

Antenna (Fig. 2D) coxa with single seta; basis fused to elongated first endopodal segment forming allobasis with eight setae on outer medial margin, and single lateral seta and transverse row of small spinules terminally; second segment with eight outer setae and fine setules along inner margin; free terminal segment short with six setae. Exopod 3-segmented, setation formula 1, 4, 3.

Mandible (Fig. 3A) gnathobase having two sharp cuspid teeth, one blunt tooth, and three small sharp teeth bordered by small spinules at the proximal end; basis with fine setules on medial outer marginal, single seta distally, and patch of small spinules on surface at midlength; first endopodal segment short with two short setae, second segment with seven setae; exopod 4-segmented, first to fourth with setation formula 1, 1, 2, 2; first segment with row of small spinules.

Maxillule (Fig. 2E) with precoxal arthrite bearing nine strong spines; coxal endite with three terminal setae; coxal epipodite with one short and eight long setae; basal exite with one terminal seta and one proximal seta; exopod 1-segmented fused with basis and bearing two long medial setae, five setae terminally, with fine spinules along outer marginal; endopod absent.

Maxilla (Fig. 3B) with syncoxal endite bearing 5, 3, 3, 2 setae; basis with one long seta; endopod with four long, one medium, and one short setae.

Maxilliped (Fig. 2F) highly reduced; syncoxa with setation formula of two long, one medium and one short seta; basis with one short strong, one long setae and row of setules along inner margin; endopod 2-segmented, with four inner spines and terminal spiniform element.

Legs 1 to 4 (Fig. 3C–F) biramous, each with 2-segmented endopod and 3-segmented exopod; coxa unarmed; second endopodal segments of leg 1 and 4 and third exopodal segment of leg 1 with row of small spinules anteriorly. Seta and spine formula as shown in Table 1.

**Table 1.** Acartia (Odontacartia) edentata sp. n. armature formula for legs 1–4, with spines and setae indicated by Roman and Arabic numerals, respectively, following Huys and Boxshall (1991).

	Coxa	Basis	Exopod segment			Endopod segment	
			1	2	3	1	2
Leg 1	0-0	0-0	1-1; I-1; 2,I,4			0-1; 1,2,3	
Leg 2	0-0	0-0	0-1; 0-1; 0,I,5			0-2; 1,2,4	
Leg 3	0-0	0-0	0-1; 0-1; 0,I,5			0-2; 1,2,4	
Leg 4	0-0	1-0	0-1; 0-1; 0,I,5			0-3; 1,2,3	

Leg 5 (Fig. 3G) symmetrical, coxae and intercoxal sclerite completely fused; basis longer than wide, outer margin with single lateral seta, slightly longer than terminal seta of exopod; exopod with knob-like projection basally, distal half spinulose.

**Figure 6.**
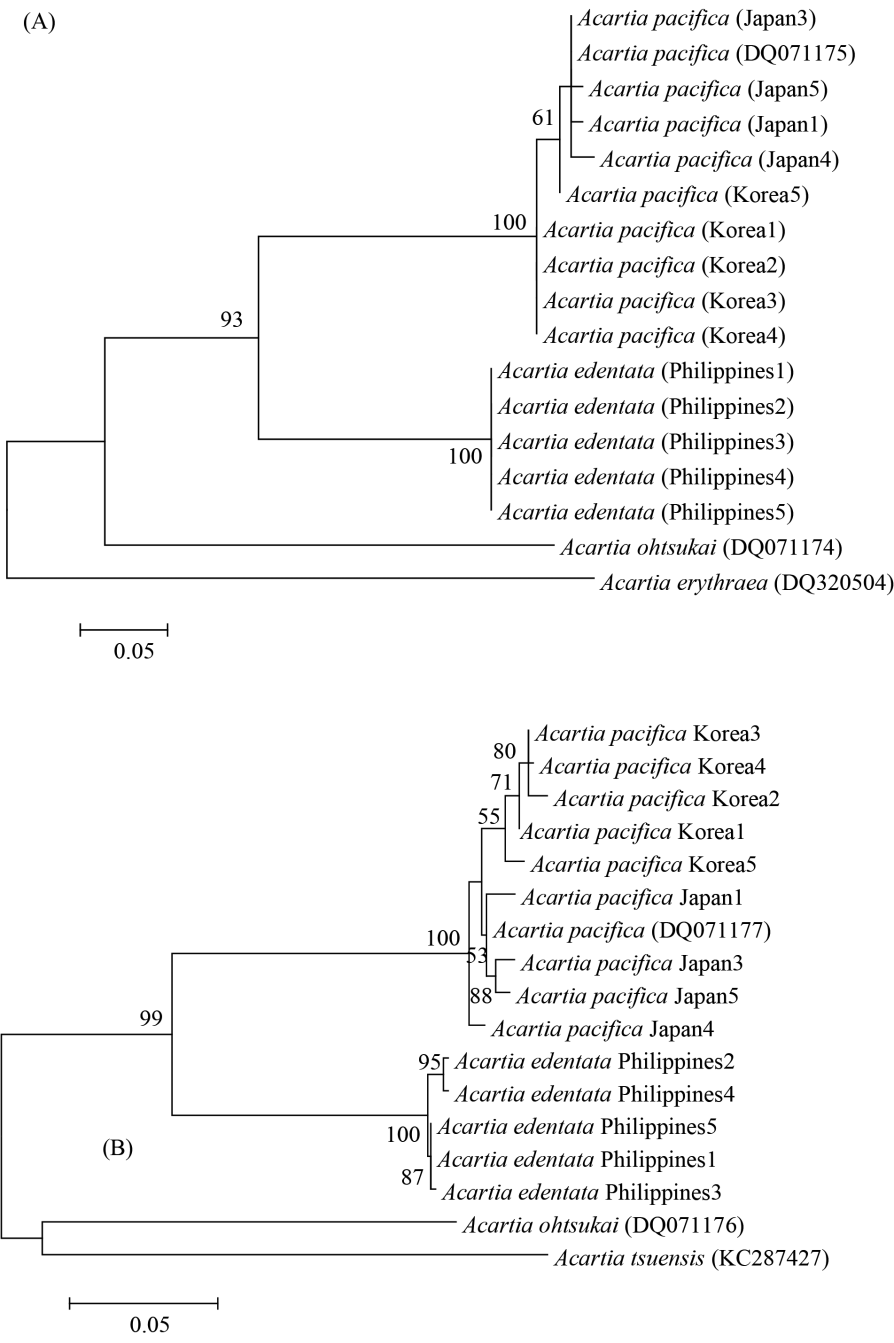
Neighbor-joining phylogenetic tree based on the mitochondrial 16S (**A**) and COI (**B**) genes of Acartia (Odontacartia) edentata sp. n., and A. (O.) pacifica from Ariake Bay in the Seto Inland Sea and Korean waters. A. (O.) erythraea, A. (O.) ohtsukai and A. (O.) tseuensis sequences from GenBank were used as outgroups. Bootstrap values (percentage) are shown for nodes with support > 50%. Supporting valves of each node obtained from 1,000 bootstrap replications.

Male. Body (Fig. 4A, B) similar to that of female; cephalosome anterior bluntly triangular in dorsal view; rostrum (Fig. 4E) with paired filaments (Figs 4E, 5E). Posterior prosome symmetrical with pair of short acute processes dorsolaterally and longer ventrolateral acute processes, with pair of small prominences between two dorsolateral processes. Posterior margin of prosome naked. Urosome composed of five somites, symmetrical in dorsal view; genital somite (= first urosomite) as long as wide, bearing 2 dorsolateral rows of small spinules; second urosomite with two pairs of strong posterior dorsolateral, processes (Figs 4A, B, 5 E), outer shorter than inner, and furnished with three rows of minute spinules ventrolaterally (Figs 4B, 5F); third urosomite with pair of strong acute processes dorsally (Figs 4 A, 5C, D); fourth urosomite 4.5 times shorter than wide and furnished with pair of small medium-sized acute processes dorsally; anal somite with setules along outer margins. Caudal rami symmetrical, approximately 1.5 times as long as wide, having lateral setules along inner margin (Fig. 5D) and 6 plumose (II–VII) setae as in female.

Left antennule (Fig. 4C) incompletely 21-segmented; segments 2, 3 and 21 incompletely fused; armature elements and fusion pattern as follows (Roman numerals represent ancestral segments): I = 1, II–V = 3 + ae, XI–IX = 4 + ae, X= 1 + (1 spiniform), XI= 2 + ae, XII = 0, XIII = 0, XIV= 1 + (1 spiniform) + ae, XV = 1, XVI = 1 + ae, XVII = 1, XVIII = 1 + ae, XIX = 1, XX= 1, XXI= 1 + ae, XXII = 1, XXIII = 1, XXIV = 2, XXV = 2 + ae, XXVI = 2, XXVII–XXVIII = 4 + ae.

Right antennule (Fig. 4D) geniculate, incompletely 17–segmented, not reaching beyond posterior end of fifth pedigerous somite; segments 2 and 3 incompletely fused; segments 2, 3, 4, 7, 9, 15, 17 each with aesthetasc (ae); segments 4, 7 and 12 each with spiniform element. Armature elements and fusion pattern as follows (Roman numerals represent ancestral segments): I = 1, II–VI = 3 + (1 minute) + ae, VII–VIII = 2 + ae, IX–XI = 2 (2 spiniforms) + ae, XII = 0, XIII = 1 minute, XIV = 1 (1 spiniform) + ae, XV = 1, XVI = 1 + ae, XVII = 1, XVIII = 1, XIX = 1 + (1 spiniform), XX = 1 + longitudinal row teeth, XXI–XXIII = 3 + longitudinal row teeth, XXIV–XXV = 3+ ae, XXVI = 2, XXVII–XXVIII = 4 + ae.

Leg 5 (Fig. 3H) uniramous, coxae unarmed and completely fused with intercoxal sclerite; each side of basis with outer plumose seta subterminally, left basis approximately 2.5 times as long as wide, with concave inner margin. Right exopod 3-segmented, first segment approximately 3 times as long as wide with single seta subterminally, second segment with small subterminal spine at mid-length of inner irregularly triangular knob, third segment curved inward with small terminal spine and small inner spine midway. Left exopod 2-segmented, first segment about 2.5 times as long as wide, second segment with long inner seta and small terminal spine.

######## SEM observation of Acartia (Odontacartia) edentata sp. n.

The absence of paired dorsal processes on the female genital double-somite and the thick rostrum were confirmed with scanning electron microscopy (Fig. 5 A, D). Paired genital slits are located at midlength and moderately separated (Fig. 5B, C). A row of setules is located along the anterior margin of each genital slit (Fig. 5C).

SEM observations of the male urosomite clearly showed fine ornamentation on the posterior border of first urosomite (Fig. 5E) and second segment furnished with three rows of minute spinules ventrolaterally (Fig. 5F). No fine setules were observed along the inner posterior margin of prosome, as described in the above descriptions of the type specimens.

######## Remarks.

The subgenus Acartia (Odontacartia) is composed of two species groups, the *centrura* and *erythraea* species groups (Steuer 1923). Acartia (O.) lilljeborgi is regarded as an intermediate type between the *centrura* and *erythraea* species groups (Steuer 1923; Ueda 1986). The *centrura* species group is defined as follows: in the female, the genital double-somite with a pair of large processes, the first antennulary segment without a large spine, relatively long caudal rami, the exopod of leg 5 with a knob situated or extending to midlength; in the male, the third and fourth urosomites have a dorsal pair of large acute processes, the first exopodal segment of left leg 5 without an outer spine.

Among the *centrura* species, the female of A. (O.) edentata sp. n. is unique in lacking paired posterior dorsolateral processes on the genital double-somite unlike those of the closely related A. (O.) pacifica (Table 2). Such an absence can also be found in females of A. (O.) bowmani from India (Abraham 1976), but the morphology of posterior prosome and fifth legs of both sexes of A. (O.) bowmani differ from that of A. (O.) edentata sp. n.: (1) posterior prosomal border of female and male rounded with one pair of medium spines and one pair of small spines dorsally, (2) posterior margin of antennule with small spines on segments 4, 5, 10, 11 and 13 in female, (3) exopod in female fifth leg bulbous basally, (4) first segment of male urosomite bilobed and with fine setules on lateral margins, (5) second segment of right exopod of male fifth leg with quadrilateral shape of inner lobe, and (6) second segment of left exopod of male fifth leg with short seta and short segment. Irrespective of the presence or absence of the dorsal processes on the female genital double-somite, females of the new species and A. (O.) pacifica share the following features: (1) moderately long caudal rami (ca 2.7 times as long as wide), (2) the presence of a basal knob on the exopod of leg 5. Males of these species are also characterized together as follows: (1) the fine setules along posterior margin of first urosomite, (2) the presence of ventrolateral rows of minute spinules on the second urosomite laterally, and (3) dorsal processes on the third urosomite twice as long as those on the fourth urosomite.

**Table 2.** Differences in morphological characteristics among Acartia (Odontacartia) edentata sp. n., A. (O.) pacifica from Japan and Korea, and A. (O.) ohtsukai.

Features	A. (O.) edentata sp. n.	A. (O.) pacifica (Japan and Korea)	A. (O.) ohtsukai*
**Female**			
Setae on segment (5) VII of right antennule	1 seta	Absent	1 seta
Paired posterior dorsolateral processes on the genital double-somite	Absent	Present	Present
Length of dorsal processes on the second urosomite relative to the posterior border of the anal somite	Reaching posterior border of anal somite	Half of length	Half of length
Length ratio of lateral to terminal setae of leg 5	1.3	2	1
Mandibular processes	1 blunt and 2 cuspidate	1 blunt covered with chitosan and 6 cuspidate	5 cuspidate
**Male**			
Length of dorsal and lateral spines on second somite	Medium	Long	Short
Presence of three rows of spinules ventrolaterally on second somite	Present	Present	Absent
Dorsal processes of third urosomite long enough to reach beyond those of fourth urosomite	Reaching	Not reaching	Not reaching
Insertion of inner seta on second exopod segment of left leg 5	Midway	Subterminal	Subterminal
Shape of medial projection on second exopodal segment of right leg 5	Irregular triangular	Rounded triangular	Quadrate

*According to Ueda and Bucklin, 2006.

Since “A. (O.) pacifica” s.l. morphologically and genetically consists of several cryptic species (Ueda and Bucklin 2006; Srinui et al. unpublished), we genetically compared specimens obtained from Japan (the Seto Inland Sea) and South Korea near the type localities to the new species (see “Molecular diversity” below). In conclusion, our Japanese and Korean specimens of A. (O.) pacifica clearly coincided with A. (O.) pacifica s.s. as morphologically/genetically redefined by Ueda and Bucklin (2006). Therefore, a comparison is made between the new species from the Philippines and A. (O.) pacifica s.s. obtained from Japan and South Korea in the present study. In addition to the absence of dorsal processes on the genital double-somite, females of the new species are distinguished from those of A. (O.) pacifica s.s. by: (1) segment 5 (VII) of right antennule with 1 seta (absent in A. (O.) pacifica s.s.), (2) dorsal processes on the second urosomite nearly reaching the posterior border of the anal somite (at most half the length of anal somite in A. (O.) pacifica s.s.), (3) length ratio of lateral seta of the basis to terminal seta of leg 5 is relatively short, about 1.3 (ca 2 in A. (O.) pacifica s.s.), (4) mandibular processes on gnathobase 1 blunt and 2 cuspidate (1 blunt and 6 cuspidate in A. (O.) pacifica s.s.) (Table 2). Males of the new species are differentiated from those of A. (O.) pacifica*s.s.* by: (1) Dorsal and lateral spines on the second somite are of medium-sized (longer in A. (O.) pacifica s.s.), (2) dorsal processes of the third urosomite are long enough to reach beyond those of the fourth urosomite (not reaching in A. (O.) pacifica s.s.), (3) terminal exopod segment of left leg 5 with an inner seta inserted midway (subterminally in A. (O.) pacifica s.s.), and (4) medial projection of second exopodal segment of right leg 5 with an inner irregularly triangular knob (rounded triangular in A. (O.) pacifica s.s. (Table 2).

Ueda and Bucklin (2006) described both left and right antenules of male A. (O.) ohtsukai, and we can compare setation in the new species as follows: Right: (3) VII–VIII–2 + ae (3 and 1 ae in A. (O.) ohtsukai), (4) IX–XI–2 (2 spiniform) + ae (plus 2 spinules in A. (O.) ohtsukai), (6) XII–1 minute (minute absent in A. (O.) ohtsukai), (7) XIV–1 (1 spiniform) + ae (plus 1 spinule in A. (O.) ohtsukai), (11) XVIII–1 (plus 1 ae in A. (O.) ohtsukai), (12) XIX–1 (spiniform) (plus longitudinal row teeth in A. (O.) ohtsukai), (14) XXI–XXIII–3 + longitudinal row teeth (longitudinal row teeth absent in A. (O.) ohtsukai), (15) XXIV–XXV–3 + ae (plus 1 spinule in A. (O.) ohtsukai). The segmentation and setation of the right antennule of A. (O.) ohtsukai are alternately interpreted as follows: 16-segmented with those of (1) I–1, (2) II–VI–4 + ae, (3) VII–VIII–3 + ae, (4) IX–XI–4 (2 spiniforms) + ae, (5) XII–unarmed, (6) XIII–unarmed, (7) XIV–2 (1 spiniform) + ae, (8) XV–1, (9) XVI–1 + ae, (10) XVII–1, (11) XVIII–1 + ae, (12) XIX–1 + process, (13) XX–1 (14) XXI–XIII–4, (15) XXIV–XXV–4 + ae, (16) XXVI–2 (17) XXVII–XXVIII–4 + ae. The segmentation and setation of the left antennule are similar in the A. (O.) edentata sp. n. and A. (O.) ohtsukai.

The taxonomy of the Indo-West Pacific A. (O.) pacifica should be revised, because the presence of several cryptic species has already been suggested by our study and others. The above-mentioned sexual dimorphic features are species-specific, and should be carefully compared among A. (O.) pacifica s.l. to resolve the issue (see Discussion).

######## Etymology.

The new species of *Acartia* was named *edentata* (Latin, meaning toothless) with reference to the absence of tooth-like processes on the posterodorsal border of the genital double-somite in females.

######## Genetic diversity.

We obtained sequence data from mitochondrial 16S and COI genes for 14 individual specimens at three sites. A 282 bp fragment of the 16S gene was analyzed for five adult female specimens from the Philippines (A. (O.) edentata sp. n.), and a 162 bp 16S fragment was analyzed for A. (O.) pacifica specimens from Ariake Bay, the Seto Inland Sea, Japan and South Korea. A 636 bp fragment of the mitochondrial COI gene was analyzed in the new species and in specimens from Japan and Korea. GenBank sequences for A. (O.) pacifica (accession number DQ071175 for 16S and DQ071177 for COI) and two out group species of subgenus A. (Odontacartia), A. (O.) ohtsukai (accession number DQ071174 for 16S and DQ071176 for COI), A. (O.) erythraea (accession number DQ320504 for 16S) and A. (O.) tsuensis (accession number KC287427 for COI), were also used for comparison. The intraspecific variation in the 16S sequences from the five A. (O.) edentata sp. n. individuals was 0%, whereas A. (O.) edentata sp. n. sequences differ from those of A. (O.) pacifica from Japanese and Korean waters, A. (O.) pacifica based on GenBank, A. (O.) ohtsukai, and A. (O.) erythraea by 20–21%, 20–21%, 28%, and 31%, respectively. The COI sequences from A. (O.) edentata sp. n. individuals differ by only 0.02–0.08%; A. (O.) pacifica (from Japan, and Korea), A. (O.) pacifica (GenBank), A. (O.) ohtsukai, and A. (O.) tsuensis sequences differ from A. (O.) edentata sp. n. COI sequences by 16–18%, 16–17%, 16–17%, 22%, and 24%, respectively (Fig. 6; Table 3).

**Table 3.** Pairwise differences for 16S and COI sequences between individual females of Acartia (Odontacartia) edentata sp. n. from Leyte Island, the Philippines; A. (O.) pacifica from Ariake Bay, Seto Inland Sea (GenBank accession no. DQ071175 for 16S and DQ071177 for COI); A. (O.) pacifica from South Korea and Japan (Seto Inland Sea); A. (O.) ohtsukai from the Rokkaku River Estuary, Ariake Bay (GenBank accession no. DQ071174 for 16S and DQ071176 for COI); A. (O.) erythraea (GenBank accession no. DQ320504 for 16S); and A. (A.) tsuensis (GenBank accession no. KC287427 for COI).

16S		1	2	3	4	5	6	7	8	9	10	11	12	13	14	15	16
1	* Acartia edentata *																
2	* Acartia edentata *	0.000															
3	* Acartia edentata *	0.000	0.000														
4	* Acartia edentata *	0.000	0.000	0.000													
5	* Acartia edentata *	0.000	0.000	0.000	0.000												
6	*Acartiapacifica* (Japan1)	**0.206**	**0.206**	**0.206**	**0.206**	**0.206**											
7	*Acartiapacifica* (Japan3)	**0.206**	**0.206**	**0.206**	**0.206**	**0.206**	0.006										
8	*Acartiapacifica* (Japan4)	**0.219**	**0.219**	**0.219**	**0.219**	**0.219**	0.019	0.013									
9	*Acartiapacifica* (Japan 5)	**0.212**	**0.212**	**0.212**	**0.212**	**0.212**	0.013	0.006	0.019								
10	*Acartiapacifica* (Korea1)	**0.200**	**0.200**	**0.200**	**0.200**	**0.200**	0.025	0.019	0.031	0.025							
11	*Acartiapacifica* (Korea2)	**0.200**	**0.200**	**0.200**	**0.200**	**0.200**	0.025	0.019	0.031	0.025	0.000						
12	*Acartiapacifica* (Korea3)	**0.200**	**0.200**	**0.200**	**0.200**	**0.200**	0.025	0.019	0.031	0.025	0.000	0.000					
13	*Acartiapacifica* (Korea4)	**0.200**	**0.200**	**0.200**	**0.200**	**0.200**	0.025	0.019	0.031	0.025	0.000	0.000	0.000				
14	*Acartiapacifica* (Korea5)	**0.212**	**0.212**	**0.212**	**0.212**	**0.212**	0.013	0.006	0.019	0.013	0.013	0.013	0.013	0.013			
15	*Acartiapacifica* (DQ 071175)	0.206	0.206	0.206	0.206	0.206	0.006	0.000	0.013	0.006	0.019	0.019	0.019	0.019	0.006		
16	*Acartiaohtsukai* (DQ071176)	0.287	0.287	0.287	0.287	0.287	0.287	0.281	0.281	0.287	0.275	0.275	0.275	0.275	0.281	0.281	
17	*Acartiaerythraea* (DQ320504)	0.313	0.313	0.313	0.313	0.313	0.356	0.350	0.338	0.350	0.344	0.344	0.344	0.344	0.350	0.350	0.331
**COI**		**1**	**2**	**3**	**4**	**5**	**6**	**7**	**8**	**9**	**10**	**11**	**12**	**13**	**14**	**15**	**16**
1	* Acartia edentata *																
2	* Acartia edentata *	0.008															
3	* Acartia edentata *	0.002	0.009														
4	* Acartia edentata *	0.008	0.003	0.009													
5	* Acartia edentata *	0.000	0.008	0.002	0.008												
6	*Acartiapacifica* (Japan1)	**0.173**	**0.178**	**0.175**	**0.178**	**0.173**											
7	*Acartiapacifica* (Japan3)	**0.176**	**0.181**	**0.178**	**0.181**	**0.176**	0.019										
8	*Acartiapacifica* (Japan4)	**0.164**	**0.165**	**0.165**	**0.165**	**0.164**	0.017	0.020									
9	*Acartiapacifica* (Japan5)	**0.175**	**0.180**	**0.176**	**0.180**	**0.175**	0.017	0.011	0.019								
10	*Acartiapacifica* (Korea1)	**0.162**	**0.167**	**0.164**	**0.167**	**0.162**	0.024	0.024	0.022	0.022							
11	*Acartiapacifica* (Korea2)	**0.165**	**0.170**	**0.167**	**0.170**	**0.165**	0.030	0.030	0.028	0.028	0.009						
12	*Acartiapacifica* (Korea3)	**0.162**	**0.167**	**0.164**	**0.167**	**0.162**	0.024	0.024	0.022	0.003	0.022	0.006					
13	*Acartiapacifica* (Korea4)	**0.164**	**0.169**	**0.165**	**0.169**	**0.164**	0.025	0.025	0.024	0.024	0.005	0.008	0.002				
14	*Acartiapacifica* (Korea5)	**0.161**	**0.165**	**0.162**	**0.165**	**0.161**	0.025	0.025	0.024	0.024	0.011	0.020	0.014	0.016			
15	*Acartiapacifica* (DQ 071177)	0.167	0.172	0.169	0.172	0.167	0.009	0.009	0.011	0.008	0.014	0.020	0.014	0.016	0.016		
16	*Acartiaohtsukai* (DQ071176)	0.220	0.220	0.222	0.220	0.220	0.241	0.241	0.233	0.239	0.231	0.235	0.231	0.233	0.235	0.231	
17	*Acartiatsuensis* (KC287427).	0.243	0.244	0.244	0.244	0.243	0.247	0.252	0.244	0.249	0.250	0.249	0.247	0.249	0.252	0.243	0.247

######## Ecology.

Temperature and salinity appear to be important factors determining the distribution and abundance of copepods. In the Indo-West Pacific, A. (O.) pacifica occurs in the tropical and subtropical zones of the Pacific and Indian oceans. In the East China Sea (subtropical zone), A. (O.) pacifica was abundant in August (salinity 15.0) in the Changjiang (Yangtze River) Estuary, China (Gao et al. 2008), while in Korean waters, A. (O.) pacifica is strictly stenohaline, occurring waters of more than 32 in salinity (Moon et al. 2008). Kang (2011) also observed the A. (O.) pacifica and A. (O.) erythraea in Korean waters with temperature ranges of 18.0–27.2 °C and 14.6–26.4 °C and salinity ranges of 21.0–32.9 and 21.0–33.7, respectively. In Japanese waters A. (O.) ohtsukai was found in the estuary of the Rokkaku River, Ariake Bay in surface waters, where water temperature was 29.0 °C and salinity was 5.0, while A. (O.) pacifica was found in waters of 26.0 °C and 33.0 in the Seto Inland Sea, Japan (Ueda and Bucklin 2006). Furthermore, A. (O.) pacifica was dominant in Moreton Bay, Queensland waters with temperature ranges above 22.0 °C and salinities ranging from 34.0 to 36.5 (Greenwood 1981).

In the tropical zone, A. (O.) edentata sp. n. specimens were collected in the Philippines during the rainy season (August 2013), when water temperature and salinity were 30.2 °C and 33.5, respectively. In contrast, A. (A.) tsuensis represents the dominant species in brackish pond water from Panay Island in central Philippines during the dry season (November – April), with salinity ranging from 14.0 to 40.0 (Golez et al. 2002).

**Figure 7.**
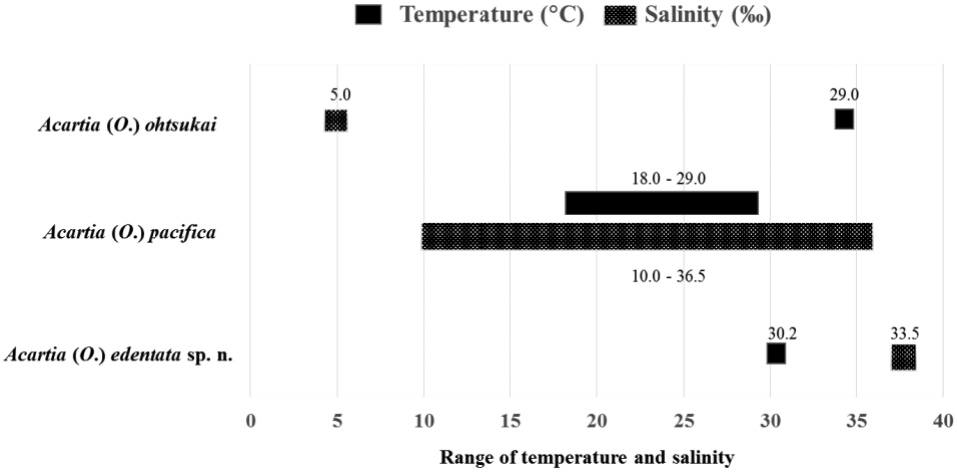
Ranges of salinity and temperature of three *Acartia* species occurring in the tropical and subtropical zones of the Pacific and Indian oceans.

In Bintulu, Sarawak, Malaysia, Johan et al. (2013) compared to A. (O.) pacifica in coastal waters with temperatures of 28.8–29.0 °C and high salinities (24.0–32.0). In the Indian waters, Wellershaus (1969) recorded the occurrence of female specimens of A. (O.) pacifica in waters with salinity ranging from 10.0 to 30.0, including in the Andaman Sea, Thailand (Surin Islands National Park, Phang Nga Province), A. (O.) pacifica were abundant in waters with temperatures of 29.7–31.0 °C and salinities ranging from 29.9 to 35.8 (Treeramaethee et al. 2013). However, we concluded that three species of *Acartia* appear to occupy water bodies differing in temperature and salinity of the tropical and subtropical zone of the Pacific and Indian oceans (Fig. 7).

## Discussion

Prior to the current study, it was believed that A. (O.) pacifica was represented by a single species with a wide geographic range occupying the coastal brackish waters throughout the Western Pacific and Indian oceans (FrÜchtl 1923; Sewell 1933; Shen and Lee 1963; Chen and Zhang 1965; Tanaka 1965; Brodsky 1967; Wellershaus 1969; Pillai 1971; Pinkaew 2003; Mulyadi 2004; Ueda and Bucklin 2006; Shang et al. 2007; Gao et al. 2008; Lan et al. 2008; Moon et al. 2008; Phukham 2008; Kang 2011; Treeramaethee et al. 2013). From the findings of the present study, A. (O.) edentata sp. n. is described based on morphological features that permit its discrimination from A. (O.) pacifica in the West Pacific Ocean. Ueda and Bucklin (2006), suggested that the features provided strong evidence specific to the habitats occupied by species and could be used to discriminate A. (O.) pacifica, which occupies neritic waters, from A. (O.) ohtsukai, which occupies brackish waters.

Mitochondrial markers within the 16S and COI genes have proved to be of great utility in investigating the systematics of ecologically and geographically isolated populations of calanoid copepods (Bucklin et al. 2003). The molecular-based analyses using 16S and COI sequences in the current study lend good support to the morphology-based findings. The findings suggest two major clades that reflect the geographic distribution of *Acartia* in the Indo-West Pacific, i.e. A. (O.) edentata sp. n. and A. (O.) pacifica from the Seto Inland Sea and from Korean waters, and A. (O.) ohtsukai from Ariake Bay, Japan. Our sequencing results agree with those of Ueda and Bucklin (2006) and emphasize the wide divergence between A. (O.) ohtsukai from A. (O.) pacifica. The high level of sequence divergence observed in this study indicates that the Philippines Islands serve as a barrier limiting the spread of A. (O.) pacifica populations into the Philippine Archipelago. This supports the allopatric speciation hypothesis of Carpenter and Springer (2005) that an ecological vicariant seems to have blocked the migration of marine organisms in the Pleistocene from the West Pacific Ocean to the Indian Ocean. Srinui and Ohtsuka (2015) showed that the distribution patterns of 11 species of *Acartiella* could be separated into those inhabiting the West Pacific and those in the Indian Ocean. In the case of A. (O.) pacifica s.l., more studies on inter- and intra-specific molecular and morphological variation found in specimens collected from Asian waters are needed to further understand the distribution and evolution of sibling species in the West Pacific region.

### Key to species of the subgenus Acartia (Odontacartia)

Thirteen species of the subgenus Acartia (Odontacartia), including A. (O.) edentata sp. n., have been described from the Indo-West Pacific (Razouls et al. 2018; present study), and are divided into three groups: the *centrura* and *erythraea* species groups and A. (O.) lilljeborgi (Steuer 1923; Ueda 1986). Key to species is provided below for both sexes of the subgenus Acartia (Odontacartia).

**Female**

**Table:** 

1	Genital double-somite lacking posterodorsal sharp processes	**2**
–	Genital double-somite having paired posterodorsal processes	**3**
2	Ventroposterior corners of prosome acutely pointed, reaching beyond half of genital double-somite	**A. (O.) edentata sp. n.**
–	Ventroposterior corners of prosome round with pair of acutely pointed processes not reaching beyond half of genital double-somite	**A. (O.) bowmani**
3	Second segment of antennule with strong curved processes posteriorly	**4**
–	Second segment of antennule without strong curved processes	**5**
4	First antennule segment with two large processes terminally	**A. (O.) bispinosa Carl, 1907**
–	First antennule segment lacking processes	**A. (O.) spinicauda**
5	Exopod of leg 5 thickened proximally	**6**
–	Exopod of leg 5 not thickened proximally	**8**
6	Exopod of leg 5 thickened proximally extending midway along exopod	**A. (O.) centrura**
–	Exopod of leg 5 with thickened proximal part confined to base of exopod	**7**
7	Length ratio of outer basal setae to exopod of leg 5: ca 2	***A. (O.) pacifica***
–	Length ratio of outer basal setae to exopod, leg 5: ca 1	**A. (O.) ohtsukai**
8	Caudal ramus longer than wide by at most ca 2 times; second free urosomite with small spinules dorsally and posteriorly	**9**
–	Caudal ramus longer than wide by ca 3 times; second free urosomite lacking small dorsal spinules	**A. (O.) mertoni**
9	Fifth to seventh antennule segments each with posterior hook; genital double-somite with two pairs of small processes dorsally	**A. (O.) lilljeborgi**
–	Fifth to seventh antennule segments each lacking hook posteriorly; genital double-somite with pair of small processes dorsally	**10**
10	First antennule segment with 2 or more strong processes distally	**11**
–	First antennule segment with single strong process distally	**12**
11	Second antennule segment with single spinule posteriorly	**A. (O.) erythraea**
–	Second antennule segment with 4 spinules posteriorly	**A. (O.) amboinensis Carl, 1907**
12	Caudal ramus with 4–6 rows of minute spinules dorsally	**A. (O.) japonica Mori, 1940**
–	Caudal ramus lacking of dorsal rows of spinules	**A. (O.) australis Farran, 1936**

**Male**

**Table:** 

1	Urosomite 3 with large spine-like processes dorsally	**2**
–	Urosomite 3 without spine-like processes dorsally	**9**
2	Dorsal processes of urosomite 3 long, reaching half-length of anal somite	**3**
–	Dorsal processes of urosomite 3 short, reaching posterior-most border of urosomite 4	**6**
3	Urosomite 4 with four spine-like processes between pair of dorsal processes	**A. (O.) spinicauda**
–	Urosomite 4 lacking spine-like processes between pair of dorsal processes	**4**
4	Genital somite lacking spinular rows along posterodorsal border	**A. (O.) pacifica**
–	Genital somite with spinular rows along posterodorsal border	**5**
5	Inner projection of first exopodal segment of right leg 5 quadrate	**A. (O.) mertoni**
–	Inner projection of first exopodal segment of right leg 5 irregularly triangular	**A. (O.) edentata sp. n.**
6	Urosomites 3 and 4 each with two prominences between pair of dorsal processes	**A. (O.) centrura**
–	Urosomites 3 and 4 each lacking prominences between pair of dorsal processes	**7**
7	Inner seta of terminal exopodal segment of left leg 5 longer than terminal segment	**A. (O.) ohtsukai**
–	Inner seta of terminal exopodal segment of left leg 5 nearly equal to terminal segment	**A. (O.) bowmani**
8	Urosomite 4 without prominences dorsally	**A. (O.) australis**
–	Urosomite 4 with prominences dorsally	**9**
9	Number of dorsal prominences on urosomite 4 fewer than five	**10**
–	Number of prominences on urosomite more than seven	**12**
10	Terminal exopodal segment of left leg 5 with three elements	**A. (O.) erythraea**
–	Terminal exoposal segment of left leg 5 with single element	**11**
11	Terminal element of left leg 5 spiniform	**A. (O.) amboinensis**
–	Terminal element of left leg 5 as fine seta	**A. (O.) lilljeborgi**
12	Terminal elements of left leg 5 as three small prominence	**A. (O.) japonica**
–	Terminal elements of left leg 5 as two spines	**A. (O.) bispinosa**

## Supplementary Material

XML Treatment for Acartia (Odontacartia) edentata
